# Hepatorenal Syndrome: direct treatment costs and characteristics of patients admitted to intensive care

**DOI:** 10.31744/einstein_journal/2025GS0390

**Published:** 2025-03-28

**Authors:** Franciele Robes Hortelã, Rodrigo Sfredo Kruger, Valéria Filomena de Oliveira, Hipolito Carraro, Dominique Araújo Muzzillo, Sérgio Candido Kowalski

**Affiliations:** 1 Postgraduate Program in Internal Medicine and Health Sciences Complexo Hospital de Clínicas Universidade Federal do Paraná Curitiba PR Brazil Postgraduate Program in Internal Medicine and Health Sciences, Complexo Hospital de Clínicas, Universidade Federal do Paraná, Curitiba, PR, Brazil.; 2 Semi-Intensive Therapy Center Complexo Hospital de Clínicas Universidade Federal do Paraná Curitiba PR Brazil Semi-Intensive Therapy Center, Complexo Hospital de Clínicas, Universidade Federal do Paraná, Curitiba, PR, Brazil.; 3 Cost Accounting Unit Complexo Hospital de Clínicas Universidade Federal do Paraná Curitiba PR Brazil Cost Accounting Unit, Complexo Hospital de Clínicas, Universidade Federal do Paraná, Curitiba, PR, Brazil.; 4 Intensive Care Unit Complexo Hospital de Clínicas Universidade Federal do Paraná Curitiba PR Brazil Intensive Care Unit, Complexo Hospital de Clínicas, Universidade Federal do Paraná, Curitiba, PR, Brazil.; 5 Department of Internal Medicine Complexo Hospital de Clínicas Universidade Federal do Paraná Curitiba PR Brazil Department of Internal Medicine, Complexo Hospital de Clínicas, Universidade Federal do Paraná, Curitiba, PR, Brazil.; 6 Department of Health Research Methods, Evidence, and Impact McMaster University Hamilton Canada Department of Health Research Methods, Evidence, and Impact, McMaster University, Hamilton, Canada.

**Keywords:** Hepatorenal Syndrome, Liver cirrhosis, Septic shock, Multiple organ failure, Health care costs, Cost of Illness, Health care economics and organizations, Direct service costs, Brazil

## Abstract

**Objective:**

The purpose of this study was to describe the direct medical costs incurred in Brazil for the treatment of Hepatorenal Syndrome in intensive care and intermediate therapy care units, and to investigate the impact of this syndrome on patient survival.

**Methods:**

This longitudinal observational retrospective study included patients with Hepatorenal Syndrome admitted to the intensive and intermediate therapy care units of a public tertiary hospital. The cost generated by each patient was the sum of the direct costs and overheads.

**Results:**

Forty-four patients with 49 episodes of suspected Hepatorenal Syndrome were analyzed, 73% male, with a mean age of 55 years (SD= 11). Diagnosis was presumed in 21 episodes (43%), because not all of the Ascites International Club’s criteria were met. Alcoholic cirrhosis was the main etiology (43%); 59% of the patients were Child-Pugh Class C at admission, with a mean (SD) model for end-stage liver disease score of 24.6 (8). Seventy-seven percent of the patients died, 32% from multiple organ failure, 29% of septic shock and 27% of hypovolemic shock. The median (IQR) of the total treatment cost for each patient was Int$14,819 (8,732-23,854). The median (IQR) length of intensive care unit stay in intensive care was 11 days (7-19). Patients with a presumed diagnosis did not have a higher hospitalization cost (p=0.249) than those with true Hepatorenal Syndrome.

**Conclusion:**

The treatment of Hepatorenal Syndrome represents a significant cost, and new resource allocation in strategic areas, such as the treatment and monitoring of patients with cirrhosis, is necessary to improve their outcomes.

## In Brief

Hepatorenal Syndrome is a potentially reversible syndrome of endstage cirrhosis. It is a severe complication of cirrhosis that involves a high mortality rate and a significant economic impact on the healthcare system. This study shows the costs of the resources used for disease management and complications to be Int$14,189. Quantifying this figure contributes to an understanding of the economic impact of Hepatorenal Syndrome on patient survival.

## Highlights

■ The treatment of Hepatorenal Syndrome places a huge economic burden on healthcare systems.

■ Cost analysis studies of Hepatorenal Syndrome management can help to rationally allocate resources in healthcare.

■ Timely diagnosis and management of Hepatorenal Syndrome may reduce mortality, resource utilization and costs.

## Graphical abstract

**Figure:**
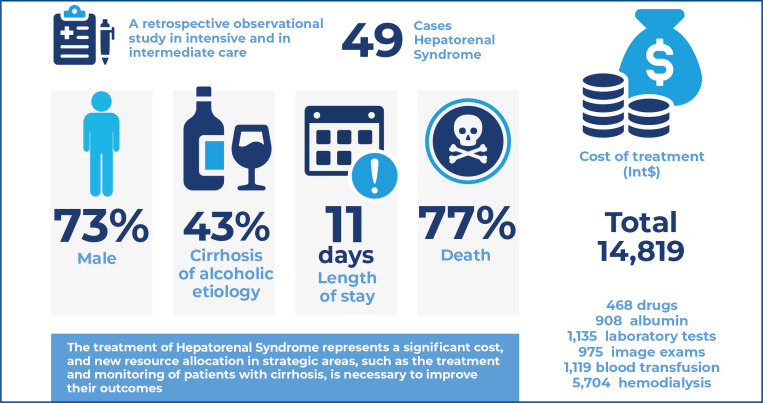

## INTRODUCTION

Hepatorenal Syndrome (HRS) is a potentially reversible syndrome that occurs in patients with cirrhosis, ascites, and hepatic failure.^1^ It is characterized by a marked reduction in renal function in the absence of other renal failure causes. Hepatorenal Syndrome can also develop in patients with circulatory dysfunction after large-volume paracentesis, acute gastrointestinal hemorrhage, or infection, particularly in cases of spontaneous bacterial peritonitis (SBP), which progresses to HRS in approximately 30% of patients.^2,3^

Treatment includes albumin infusion associated with vasopressors; in more severe cases, the patient may undergo dialysis, transjugular intrahepatic portosystemic shunt (TIPS), and even liver transplantation.^4,5^Patients are generally better managed in an intensive care unit (ICU), mainly when norepinephrine is used.^3,6^Hepatorenal Syndrome accounts for approximately 11% of acute kidney injury in hospitalized cirrhotic patients with refractory ascites.^7^ Patients with HRS have a 15% survival probability 3 months after hospitalization; their length of stay (LOS) is longer than that of patients without HRS, and the cost of hospitalization is higher.^8,9^

Considering the high level of expenditure in the healthcare system, health economics can present resource (often limited) allocation strategies and has become an important tool in decision-making.^10,11^Part of health economics evaluation, the cost of illness is an empirical approach to estimating the social impact of illnesses.^11^ It includes direct, indirect, and intangible costs with economic impact on society. This information is useful for managers to understand the total expenses and their distribution by cost categories.^10,11^

The cost of HRS treatment varies globally. In the Brazilian public health system´s economic evaluation, the cost of treatment ranged from $7,500-$15,000 for about 8 days. In the United States of America (USA), the mean price was $91,504 for about 30 days, with direct medical costs expenditure being around 3 to 3.8 million per year.^6,12,13^

Primary cost drivers for patients with HRS include LOS, hemodialysis, and mortality.^12^ Intensive treatment and the costs of procedures are the main causes for high hospital cost, when compared to patients without HRS.^9^

## OBJECTIVE

Therefore, to describe data on the patient’s profile and hospitalization costs with a significant impact on the health system, we investigate the costs involved in the treatment of Hepatorenal Syndrome in a university hospital intensive care unit and intermediate care unit and the impact of Hepatorenal Syndrome on patient survival.

## METHODS

### Study population

This retrospective observational study included patients admitted in the ICU and IMCU of a public tertiary hospital in Curitiba, the capital of the state of Paraná, Brazil. The inclusion criteria were patients older than 18 years diagnosed with HRS, as defined by the International Ascites Club of 2007, or with presumed HRS diagnosed by a physician when not all diagnostic criteria could be fulfilled. The exclusion criteria were patients with incomplete medical records or patients with chronic kidney disease.

Hepatorenal Syndrome was defined according to the International Ascites Club criteria (2007):^14^as (i) cirrhosis with ascites; (ii) serum Cr >1.5mg/dl; (iii) no improvement of serum creatinine (decrease to a level of ≤1.5mg/dl) after at least 2 days of diuretic withdrawal and volume expansion with albumin; (iv) absence of shock; (v) no current or recent treatment with nephrotoxic drugs; and (vi) absence of parenchymal kidney disease as indicated by proteinuria >500 mg/day, microhaematuria (>50 red blood cells per high power field) and/or abnormal renal ultrasonography.

The study was previously approved by the hospital’s Human Ethics Committee of *Hospital de Clínicas, Universidade Federal do Paraná*, CAAE: 85049818.3.0000.0096; # 2.581.879.

### Selection

A total of 358 patients with cirrhosis and HRS were consecutively enrolled in the study between January 1, 2014 and February 28, 2018.

Initially, the sample was consecutively selected from the hospital´s database of patients diagnosed with liver cirrhosis, upper digestive bleeding, acute renal failure, or HRS. Electronic medical records were used to separate cirrhosis patients with creatinine levels higher than 1.5mg/dl.

### Data collection and cost analysis

Data were collected from medical charts by the researcher (FR) using a semi-structured tool developed with a basis on previous expert opinions.^15^ It included basic demographics (age and sex) and data from the patient’s treatment history, including drugs and comorbidities, routine blood tests, risk scores, LOS, and complications during ICU and IMCU LOS. The cost analysis considers the hospital’s perspective.

The hospital´s Accounting and Cost Unit reported the monetary value of drugs, laboratory and imaging tests, hemodialysis sessions, and blood component bags. Individual costs were multiplied by the number of times the patient needed those services/drugs. The Accounting and Cost Unit also reported the costs of hospital ICU, IMCU, and surgical ICU fees for the year 2016.

The absorption apportionment methodology was used to transfer the daily rates.^16^ The hospital’s activities were subdivided into services, sections or units to which resources were allocated. The cost of all services used in ICU and IMCU was considered through specific apportionment.

Intensive care unit and IMCU rates comprised direct costs, overheads and apportionment.

Direct costs included salaries and materials (hospital medical supplies, kitchen supplies, hygiene, maintenance, and medical gases). Overheads were spent on outsourced services, water, electricity, and sewage. Apportionments from various sectors involved all the hospitalization units mentioned, such as management, clinical engineering service, dietetic nutrition, clothing, information technology, transportation, accounting, epidemiology, laundry, and legal sectors, among others (Table 1S, Supplementary Material).

Daily rates were calculated by dividing the monthly costs by the number of patient-days in the month (Table 1S, Supplementary Material). The daily rates consisted of salaries, materials and apportionments as mentioned above. The costs of drugs, procedures, and examinations were excluded from the daily rates, as they were calculated individually per patient.

The cost of hospitalization in the above-mentioned care units was obtained by adding the daily rates to the costs of examinations, blood transfusion, hemodialysis, and drugs used by the patient.

The costs are presented in international dollars (Int$). Conversion from Brazilian Reais to international dollars was performed using the purchasing power parity conversion factor provided by the World Bank in 2016.^17^

### Statistical analysis

The results were presented as frequencies, measures of central tendency, and dispersion. To compare the direct cost of treatment of patients with all diagnostic criteria versus those with a presumed diagnosis, the Kolmogorov-Smirnov test was used to check the normality of the data, and the Mann-Whitney U test was used to compare the two groups. Statistical analysis was performed using the SPSS version 22.0 (IBM, Armonk, NY, USA), and results with p<0.05 were considered statistically significant.

## RESULTS

**Figure 1:**
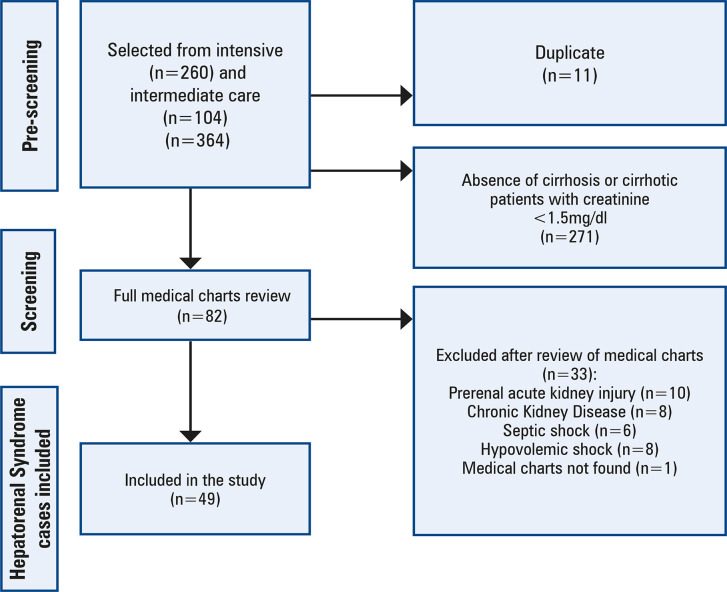
Patients selected for the study

Three hundred sixty-four patients were pre-screened for HRS after review of the electronic medical charts and creatinine levels. In the pre-screening, 11 duplicate and 271 patients not diagnosed with cirrhosis or cirrhotic patients with creatinine <1.5mg/dl were excluded. Then, 82 patients were included for the second screening phase, which involved a medical charts review. Thirty-three were excluded because they had organic kidney disease or circulatory shock. In all, 44 patients were included; five of them with two hospitalization periods, consequently, 49 cases of HRS were included in the study (Figure 1).

The demographics and clinical characteristics of the patients included are shown in table 1 as follows: The majority were male (73%) with cirrhosis of alcoholic etiology (43%). Type 1 HRS occurred in 78% of cases. Almost half of the patients (44%) had a risk factor for HRS; the most frequent was upper gastrointestinal bleeding (UGB) (20%) followed by SBP in 18%. Two patients had combined risk factors (UGB + SBE). Most frequent hospital complications were encephalopathy (78%) followed by bacterial infectious disease (69%). Twenty-five percent of our patients required hemodialysis, 85% of the sessions were conventional intermittent hemodialysis (IHD), and 15% of them were low efficiency hemodialysis (SLED).

**Table 1:** Demographic data and clinical characteristics of patients with Hepatorenal Syndrome

Variables	Results (%)*		
	Survivors (n=15)	No survivors (n=34)	All (n=49)
Age, Years^†^, n (%)	57 (10)	55 (11)	55 (11)
Male, n (%)	11 (73)	25 (73)	36 (73)
Systemic arterial hypertension, n (%)	8 (53)	8 (23)	16 (33)
*Diabetes mellitus*, n (%)	5 (33)	7 (21)	12 (25)
Cardiovascular disease, n (%)	1 (7)	3 (9)	4 (8)
Active smoking, n (%)	3 (20)	8 (23)	11 (22)
Active alcoholism, n (%)	2 (13)	7 (21)	9 (18)
Cirrhosis etiology, n (%)			
Alcoholic liver disease	7 (47)	14 (41)	21 (43)
Hepatitis C Virus	2 (13)	6 (18)	8 (16)
Hepatitis B Virus	2 (13)	3 (9)	5 (11)
Non alcoholic steatohepatitis	2 (13)	2 (6)	4 (8)
Autoimmune hepatitis	1 (7)	2 (6)	3 (6)
Hepatitis C Virus + alcoholic liver disease		2 (6)	2 (4)
Hepatitis B Virus + alcoholic liver disease		1 (2)	1 (2)
Hepatitis C Virus + hepatocellular carcinoma	1 (2)		1 (2)
Schistosomiasis		1(3)	1 (2)
Budd Chiari		1 (3)	1 (2)
Drugs		1 (3)	1 (2)
Cryptogenic		1 (3)	1 (2)
HRS type, n (%)			
Type 1	10 (67)	8 (82)	38 (78)
Type 2	5 (33)	6 (18)	11 (22)
Risk Scores^£^, n (%)			
APACHE II	15 (13 -17)	20 (16 - 23)	18 (15 - 21)
MELD admission	20 (15 - 24)	26 (21 -32)	25 (20 - 31)
Precipitants to HRS	5 (33)	17 (50)	22 (45)
Upper gastrointestinal hemorrhage	3 (20)	7 (21)	10 (20)
Spontaneous bacterial peritonitis	2 (13)	7 (21)	9 (18)
Paracentesis >5L without albumin replacement	1 (7)	5 (15)	6 (12)
Complications, n (%)			
Hemodyalisis	3 (20)	9 (26)	12 (25)
Mechanical ventilation	3 (20)	26 (76)	29 (59)
Encephalopathy	6 (40)	30 (88)	36 (74)
Spontaneous bacterial peritonitis	3 (20)	7 (21)	10 (20)
Upper gastrointestinal hemorrhage	3 (20)	14 (41)	17 (35)
Transjugular intrahepatic portosystemic shunt		3 (9)	3 (6)
Bacterial infection^§^	8 (53)	26 (76)	34 (69)
Transfusion	4 (27)	23 (68)	27 (55)
Liver transplantation	1 (7)	1 (3)	2 (4)
LOS in Hospital, days^£^	26 (19 - 41)	16 (10 - 22)	18 (11 - 27)
LOS in ICU^‡^	11 (5 - 18)	7 (4 - 10)	10 (4 - 14)
LOS in IMCU^≠^	9 (5 - 18)	12 (8 - 20)	7 (5 - 20)
LOS in ICU and IMCU	9 (5 - 20)	12 (8 - 19)	11 (7 - 19)

* Rounded to one digit; † Data are mean (SD); ^£^ Data are median (IQR); § Except spontaneous bacterial peritonitis; ‡ Median (IQR) of 21 patients, 4 survivors and 17 no survivors; ≠ Median (IQR) of 37 patients, 13 survivors and 24 no survivors.

HRS: Hepatorenal Syndrome; ICU: intensive care unit; IMCU: intermediate care unit; LOS: length of stay.

### Risk scores

The models for end-stage liver disease (MELD) by HRS type were 26 for type-1 and 19 for type-2. Most patients had Child-Pugh Class C (CP-C) on admission (59%). In some cases, the score could not be calculated because of the absence of data in the electronic medical charts or examinations required for measurement. Additional data are presented in Table 2S, Supplementary Material.

### Outcomes

Table 2 shows the clinical outcomes of patients. Seventy seven of patients died. Two patients underwent cadaveric donor liver transplantation. Both patients underwent hemodialysis during inpatient admission for HRS treatment. One of them (CP-C, MELD 19) had cirrhosis caused by the hepatitis *C virus*, waited 2 months for liver transplantation, and died the day after surgery. The other patient had alcoholic cirrhosis (CP-B, MELD 20), was transplanted during inpatient admission, and was discharged approximately 1 month later, no longer requiring hemodialysis.

**Table 2:** Causes of death of patients with Hepatorenal Syndrome

Variables	Results (%)*
Causes of death	
Multiple organ failure	11 (32)
Septic shock	10 (29)
Hypovolemic shock	9 (27)
Acute respiratory insufficiency	1 (3)
TIPS postoperative	1 (3)
Postoperative liver transplantation	1 (3)
Cerebral intraparenquimal hemorrhage	1 (3)
Mortality associated with HRS type	
Type 1	28 (74)
Type 2	6 (56)

n=34. *Rounded to one digit.

HRS: Hepatorenal Syndrome.

In 21 patients (43%), HRS was presumed because not all diagnostic criteria were met. The median (IQR) ICU or IMCU LOS was 13 days (range, 9-20 days) for patients with a defined diagnosis, and 10 days (range, 4-17 days) for those with a presumed HRS. Those patients that passed away with a defined diagnosis represented 71% in the first group and 67% in the second group. Regarding the prognosis scores, in the first group, the mean (SD) MELD score was 25.2 (7), the APACHE II was 17.6 (6), with 67.9% of the patients being CP-C on admission. In the second group, the MELD score was 23.5 (9) and the APACHE II was 19.5 (6), with 47.6% of patients being CP-C on admission.

### Resources and costs

The median (IQR) cost of admission to the ICU and IMCU was Int$14,819 (8,732-23,854) (Table 3). The median (IQR) cost of hemodialysis (n=12) was Int$5,704 (3,259-11,408), and accounted for most of the hospitalization cost. The mean (SD) of cost of salaries, hospital medical supplies, medical gases and telephone, electricity, sewage, and outsourced services was Int$4,314 (135); Int$302 (6.90), Int$1.86 (0.10) and Int$93 (4.24), respectively. The cost of liver transplantation were not included.

**Table 3:** Costs of Hepatorenal Syndrome treatment in intensive care unit and intermediate care unit

Resources	Median (IQR) Costs in International Dollars (Int$)* per length of stay		
	Survivors (n=15^**£**^)	No survivors (n=34)^†^	All (n=49)^‡^
Drugs	280 (83 - 519)	603 (405 - 1,505)	468 (275 - 1,146)
Albumin	655 (429 - 1,336)	1,008 (466 - 1,840)	908 (403 - 1,614)
Laboratory tests	599 (412 - 1,058)	1,319 (970 - 2,589)	1,135 (600 - 2,049)
Image Exams	995 (173 - 1,159)	705 (177 - 1,220)	975 (173 -1,188)
Blood Transfusion	466 (373 - 979)	1,306 (746 - 3,795)	1,119 (560 - 3,129)
Hemodialysis	11,408 (9,778 - 11,408)	4,889 (3,259 - 6,519)	5,704 (3,259 - 11,408)
ICU and IMCU Costs per LOS	12,635 (5,850 - 17,095)	17,741 (9,739 - 24,365)	14,819 (8,732 - 23,854)
ICU and IMCU Costs per patient day	1,058 (876 - 1,246)	1,395 (1,135 - 1,648)	1,224 (993 - 1,579)

* Cost based on the year 2016; ^£^Median (IQR) of 14 patients who used albumin, 13 who had image exams, 4 who had blood transfusion and 3 who underwent hemodialysis; ^†^ Median (IQR) of 23 patients who had blood transfusion and 9 who underwent hemodialysis; ^‡^Median (IQR) of 48 patients who used albumin, 47 who had image exams, 27 who had blood transfusion and 12 who underwent hemodialysis.

ICU: intensive care unit; IMCU: intermediate care unit; LOS: length of *stay.*

Regarding the use of vasopressors and ICU and IMCU admission cost, the mean (SD) costs were Int$19,586 (15,600) for noradrenaline (n=26); Int$27,336 (18,867) for noradrenaline and terlipressin (n=4); Int$18,141 (13,697) noradrenaline and octreotide (n=12); Int$20,909 (9,342) for noradrenaline, terlipressin and octreotide (n=3); Int$11,645 (9,471) for octreotide use alone (n=2); and Int$7,104 (2,562) without any drug use (n=2). For 4 out of the 7 patients who received terlipressin, the cost accounted for approximately 75% of the total drug cost (excluding the albumin cost).

The cost of treatment with albumin (p=0.101) and total cost of inpatient admission (p=0.249) showed no significant difference.

## DISCUSSION

Hepatorenal Syndrome is a serious complication of end-stage cirrhosis, caused by extreme circulatory dysfunction. Notwithstanding treatment, without a liver transplant, the 3-month mortality rate remains high and places a significant economic burden on the health care system.^2,12,18^ This is the first observational study that describes the cost components of HRS in Brazil, highlighting the economic impact of the disease. Forty-four patients, 73% male with a mean age of 55 years were analyzed. The median (IQR) length of ICU stay in intensive care was 11 days (7-19). The median (IQR) of the total treatment cost was Int$14, 819 (8,732-23,854).

In Brazil, the Unified Health System (SUS - *Sistema Único de Saúde*), offers comprehensive and totally free healthcare to the entire population and, in 2016, it paid about Int$216 per day for a daily ICU stay at CHC-PR.^19^ However, our data showed that the costs incurred were much higher (Int$1,224) than the amounts paid by the SUS, corroborating the high costs involved in treating HRS and the consequent financial *deficit*.

In the United States, a retrospective study reported a mortality rate of 36.9 % in 2,542 patients.^12^ By contrast, close to 80% of patients in our study passed away, although this represents only the very clinically ill patients who were admitted to the ICU or IMCU. Our findings are consistent with those of a meta-analysis by Weil et al., where patients with cirrhosis had a mortality rate in the ICU ranging from approximately 35% to 72%.^20^ Our patients were hospitalized in a very serious condition, a fact verified by the prognostic scores (59% of the patients were CP-C on admission), which justifies the worst evolution and is consistent with previous results in the literature that show unfavorable evolution of the disease.

Treatment is based on the use of vasoconstrictors and a high-cost infusion of albumin,^6^ substantially impacting the economy in terms of direct costs. This highlights the need for accurate prescriptions, as despite the cost, failing to correctly prescribe these medications can have serious consequences for the patient.

Large-volume paracentesis, without albumin replacement, occurred in 12% of our patients, and the lack of replacement was identified as a precipitating factor for HRS in these patients. At this stage, the possibly unnecessary pain and suffering caused by this, is impossible to calculate. In addition to these personal consequences, high direct costs to the health system may have been avoided.

The severe clinical condition of patients with HRS, require high-cost therapies. According to the EASL guidelines^3^ these patients are better managed in ICUs. IMCUs are suitable for critically ill patients, who are less critical than those admitted to the ICU, and more severe than those admitted to the ward. An IMCU cost is lower than an ICU one, provided that the patient spends less time in the intermediate unit than in the ICU.^21^ In our study, the median (IQR) IMCU LOS was 10 days (5-20 days), which was higher than the 7 days (4-14) in the ICU.

Jamil et al. showed that for 181 patients admitted to the ICU in the United States, the mean cost was US$78,770 per patient, with a hospital LOS of 5.25 days. This amount was spent on high-cost hospital procedures (blood transfusion, mechanical ventilation, dialysis, TIPS, paracentesis, and combined liver and kidney transplants), with mortality, LOS, and dialysis associated with the cost of HRS.^12^ In our study, the direct costs were lower, but this was with a smaller number of patients and without considering the costs of mechanical ventilation and transplantation, which accounted for the added high costs in the aforementioned study.

In France, Levesque et al. demonstrated that patients with cirrhosis admitted to the ICU and requiring mechanical ventilation had a worse prognosis, with a mortality rate of approximately 66% in the 246 patients evaluated.^22^ Likewise, almost 60% of our patients required mechanical ventilation, indicating the severity of their clinical condition and contributing to the high mortality rate.

The resources most frequently used during hospitalization are hemodialysis, blood transfusions, vasopressors, and albumin infusion. Renal dysfunction resulting from HRS and the presence of complications such as digestive bleeding made it necessary to use such resources. Twenty-five percent of our patients underwent hemodialysis and the cost of this procedure reached almost half of the total hospitalization cost (38%).

In our study, 27 patients required blood transfusions and almost half (48%) requered to treat UGB. The need to use blood products, which are expensive resources, demonstrates how complications that occur during hospitalization can increase the final direct cost. Vasopressors also influenced this total cost.

A Brazilian study^6^ based on hypothetical SUS hospitalizations of approximately 8 days duration, estimated costs, including depreciation rate, maintenance, salaries, equipment, and medication purchase. They found them to be Int$7,437 with terlipressin and Int$8,406 with norepinephrine, indicating that the costs of norepinephrine were higher due to the need to use an ICU bed. They argued that HRS treatment with terlipressin should be considered as a way of lowering the number of occupied ICU beds, given their universal shortage. In the aforementioned study, it was possible to avoid the costs associated with treatments, infections, and UGB.^6^

In India, Singh et al. prospectively reported the exclusive costs of using vasoconstrictors (Int$364 with noradrenaline; Int$1,291 with terlipressin) in 60 patients during 15 days of treatment.^13^ However, his study aimed primarily to evaluate the effectiveness and safety of norepinephrine compared to terlipressin. Without estimating the costs of ICU admission, they concluded that the use of norepinephrine was comparable to that of terlipressin and was less expensive.

In our study, approximately 50% of the patients were treated only with norepinephrine, and only seven of them used terlipressin, a medication with a very high hospital cost in Brazil. Of note, is that the four patients who used terlipressin, the cost accounted for approximately 75% of the total cost of medications (except for albumin), demonstrating the impact of the cost of this drug in the final expense calculation.

In our study, the mean (SD) cost of treatment with norepinephrine was Int$19,586 (15,600), which is much higher than that in the Indian study. However, our study included all expenses incurred in the IMCU and ICU hospitalizations. Notably, this drug was not used to treat HRS alone, but also to improve the patient’s hemodynamic condition.

We also investigated whether the treatment of patients with presumed HRS (not meeting all diagnostic criteria) would incur a higher albumin cost than those with a confirmed diagnosis. The results were not statistically significant (p=0.101). In our study, 43% of the cases were presumed to be the same as the 36% described by Salerno et al.^23^ They also reported clinical similarities between these groups and suggested that presumed HRS could be considered true HRS and should be treated in the same way.

In the study by Jamil et al. (n=2,542), the APACHE II score ranged between 15 and 19 in 36.3% of patients, and the overall mortality rate was 36.9%.^12^ In our study, the mean (SD) was 18^6^ leading to an expected mortality rate of 24%, which did not correspond to our reality of almost 80% mortality. The study that validated this score^24^ did not involve patients with liver disease; therefore, it was difficult to predict the risk of death in patients with cirrhosis. The APACHE score is a good indicator of severity; however, it may not accurately estimate the mortality rate.

This was a retrospective evaluation of a small number of patients with a rare disease, and data were collected from only one study center. Therefore, this study has some limitations owing to the lack of adequate information. Additionally, some physical charts are illegible and lack important data, resulting in charts with limited information on what actually happened at the bedside.

Patients with HRS have poor prognosis. Thus, the development of new diagnostic strategies and tools can facilitate early disease recognition and rapid treatment, which could contribute to lower mortality rates. Furthermore, establishing protocols for the treatment of disease risk factors could help prevent HRS, patients with decompensated cirrhosis and their families should be contacted in advance to discuss palliative care for effective special care planning and treatment.

Considering the high costs of the health system, the rational use of resources is essential for strategic disease management. Cost analysis and the resulting improved resource allocation can lead to better decision-making in the health sector, where resources are scarce.^25^

The treatment of HRS requires many resources that imply high costs for the hospital. Our study included hemodialysis, blood transfusion, drugs, laboratory, and imaging tests. However, more effective strategies could include early treatment of patients with cirrhosis, better planning and saving resources for liver transplantation, and new renal replacement therapies are actions that could have been introduced and would have reduced the long-term cost of the disease.

## CONCLUSION

Our results contribute to the development of new treatment strategies to avoid unnecessary resource consumption and achieve better results at a lower cost. Moreover, new healthcare policies can contribute to the rational allocation of resources when managing cirrhosis, to improve the prognosis of these patients.

Hepatorenal Syndrome is a severe complication of cirrhosis that has a high mortality rate and a significant economic impact on healthcare systems. Larger studies are required to provide a detailed assessment of costs and help in the decision-making process for cost-effective treatments.
