# Supplementary material for: Hepatorenal Syndrome: direct treatment costs and characteristics of patients admitted to intensive care
Source: Einstein (Sao Paulo). 2025 Mar 28;23:eGS0390. doi: 10.31744/einstein_journal/2025GS0390 (PMC12014156; doi:10.31744/einstein_journal/2025GS0390)
Supplement: Supplementary file 1 [file 2317-6385-eins-23-eGS0390-suppl01.pdf]

SUPPLEMENTARY MATERIAL

Hepatorenal Syndrome: direct treatment costs and characteristics of patients admitted to intensive care

Franciele Robes Hortelã, Rodrigo Sfredo Kruger, Valéria Filomena de Oliveira, Hipólito Carraro Junior, Dominique Araújo Muzzillo[5], Sérgio Candido Kowalski

DOI: 10.31744/einstein_journal/2025GS0390

Table 1S. Intensive care unit and intermediate care unit cost per patient day - 2016

Direct costs	ICU		Surgical ICU		IMCU	
	Monthly mean (Int$)	SD (Int$)	Monthly mean (Int$)	SD (Int$)	Monthly mean (Int$)	SD (Int$)
Health professionals						
MEC salaries	113,958.28	32,178.35	0.00	0.00	108,540.01	2,1379.39
MEC benefits	9,451.39	2,209.44	0.00	0.00	11,651.33	2,021.18
FUNPAR salaries	18,901.89	2,664.41	3,366.77	929.00	8,792.71	1,236.58
FUNPAR social charges	5,231.55	633.11	949.98	294.80	2,436.10	336.62
FUNPAR benefits	2.392.79	252.07	232.26	108.78	773.81	37.47
MEC medical salaries	67,473.21	16,192.61	0.00	0.00	3,243.30	947.45
MEC medical benefits	3,416.55	450.87	0.00	0.00	218.04	11.50
Teachers	7,326.99	2,334.47	0.00	0.00	0.00	0.00
Residents	6,094.39	352.99	0.00	0.00	8,696.70	1,568.52
Total salaries	234,247.05	48,954.50	4,549.01	1.332.57	144,352.00	21,966.13
Materials						
Hospital medical supplies	13,946.39	4,064.69	5,525.45	1.127.45	12,273.90	2,740.08
Medicinal gases	107.22	43.13	30.78	23.30	59.86	33.74
Kitchen supplies	7.58	3.91	4.02	3.11	9.94	5.23
Hygieneand cleaning material	2,602.03	474.66	1,353.99	379.90	2,273.46	395.46
Maintenance material/works	477.96	462.40	26.89	31.17	315.59	335.66
Office supplies	191.82	72.00	104.91	22.73	202.03	48.13
Other materials	101.23	105.70	48.28	18.24	91.35	45.87
Total	17,434.22	4,164.17	7,094.33	1.177.07	15,226.14	2,734.16
Overhead costs						
Third party services	2,405.67	2,357.44	0.00	0.00	2,636.36	2,641.14
Water/sewage	743.47	106.18	513.15	221.09	745.11	106.42
Electric power	984.07	217.06	1,026.97	219.48	985.71	216.25
Telephone	18.55	5.61	0.00	0.00	16.72	9.09
Total	4,151.76	2,456.55	1,540.12	401.75	4,383.89	2,743.19

continue...

...Continuation

Table 1S. Intensive care unit and intermediate care unit cost per patient day - 2016

Direct costs	ICU		Surgical ICU		IMCU	
	Monthly mean (Int$)	SD (Int$)	Monthly mean (Int$)	SD (Int$)	Monthly mean (Int$)	SD (Int$)
Apportionment received						
Board	5,698.45	842.98	562.56	111.74	4,155.49	749.92
Administrative Activities	1,379.06	536.71	136.01	59.04	1,020.97	433.72
People Development Unit	544.32	139.64	11.13	2.38	439.47	132.76
Pipa Encantada Educational Center	2,768.39	207.07	0.00	0.00	3,460.49	258.83
Personnel Administration Service	1,070.60	156.16	22.89	8.28	860.00	153.01
Service Hospital Billing	1,600.64	511.43	447.48	304.13	1,526.70	755.18
Security Service and Traffic Control	2,128.64	351.67	2,166.04	357.85	4,674.78	772.31
Telephony Section	220.00	35.81	0.00	0.00	165.00	26.85
Reprography Section	17.57	16.74	0.00	0.00	3.32	6.09
Computer Service	1,497.52	433.70	0.00	0.00	1,497.52	433.70
Transport Service	0.00	0.00	0.00	0.00	7.16	13.17
Medical File Service	153.98	160.52	217.16	32.61	471.35	121.88
Operational Support Service for Hospitalization	3,286.99	3,068.00	549.99	313.22	4,844.74	576.23
Hospital Supplies Service	16,771.77	17,536.81	10,809.01	7.000.05	23,833.78	18,243.67
Hospital Hygiene Service	14,237.23	6,364.22	0.00	0.00	20,428.91	3,043.93
Boiler Team	5,880.52	4,483.75	583.02	367.50	2,096.42	388.89
Nutritionand Dietetics Service	3,291.61	1,982.13	3,531.55	1.485.47	12,922.36	6,577.67
Anesthesiology Section	3,246.21	2,792.81	651.18	1.312.62	79.47	236.79
Hospital Engineering and Maintenance Service	2,805.92	2,573.35	0.00	0.00	1,604.51	1,326.11
Clinical Engineering Service	6,495.59	1,861.21	0.00	0.00	5,135.57	1,442.09
Clothes Making Service	938.72	335.67	0.00	0.00	2,127.16	777.24
Laundry Service	8,615.91	4675.28	0.00	0.00	17,831.06	4,978.97
Sterilized Materials Processing Unit	6,467.04	1,748.18	2,506.02	739.66	4,836.88	897.82
Hospital Pharmacy Service	10,886.30	3,488.72	3,400.98	1.072.82	15,286.85	11,117.11
Hospital Infection Control Service	2,863.23	672.92	0.00	0.00	1,041.80	244.84
Social Service	5,508.06	3,191.62	1,155.83	734.49	5,415.81	4,157.51
Medical Residence	420.71	206.03	0.00	0.00	420.71	206.03
Medical Gas Team	6,242.94	1,804.76	0.00	0.00	4,776.79	1,380.91
Accounting and Finance	1,468.13	208.79	145.24	29.00	1,077.57	228.05
Specialized Services in Safety Engineering and Occupational Medicine	214.79	28.58	4.51	1.39	172.50	27.45
Cost Accounting Unit	693.26	78.11	68.80	14.14	504.79	64.72
Human Resources assessment and monitoring service	733.52	174.42	16.37	8.60	582.81	120.10
Epidemiology Service	2,299.56	602.56	224.29	52.52	1,653.18	324.56
Protocol Section	357.02	67.84	35.05	7.43	259.19	48.82
Ombudsman Service	257.20	79.95	25.06	6.40	189.12	70.55
Projects and Supervision of Works and Renovations Section	646.87	121.68	63.16	10.68	470.34	95.26
Head of the Urgency and Emergency Unit	7,498.84	1,925.95	7,498.84	1.925.95	7,498.84	1,925.95
Dressing Room Team	221.53	27.70	4.77	1.79	177.34	23.21
Enteral and Lactation Nutrition Section	13,002.14	2,931.86	1,787.54	810.42	8,320.23	1,781.49
Total apportionments	142,430.78	17,747.49	36,624.49	7.568.02	161,871.00	28,750.75
Total direct costs + overhead + apportionments	398,263.82	57,627.07	49,807.94	7.673.91	325,833.03	43,201.61
Day-atients	461	14	193	12	12	53
Daily cost	867.47	144.05	259.94	53.56	631.52	121.09

ICU: intensive care unit; IMCU: intermediate care unit; Int$: international dollars; MEC: Ministry of Education, Brazil; FUNPAR: Foundation to support the *Universidade Federal do Paraná*.

Table 2S. Meld, Child and APACHE II scores of patients with Hepatorenal Syndrome

Variables	Results (%)*		
	Survivors (n=15)	Nonsurvivors (n=34)	All (n=49)
MELD score			
Admission			
10 -19	6 (40)	5 (15)	11 (23)
20 -29	6 (40)	16 (47)	22 (45)
30 - 39	1 (7)	11 (32)	12 (25)
40		1 (3)	1 (2)
Undetermined	2 (13)	1 (3)	3 (6)
Outcome	20 (6)**	33 (9)**	30 (9)**
Undetermined	10 (67)	14 (41)	24 (49)
APACHE II score			
5 - 9	1 (7)	1 (3)	2 (4)
10 - 14	3 (20)	3 (9)	6 (12)
15 - 19	10 (66)	11 (32)	21 (43)
20 - 24		10 (29)	10 (21)
25 - 29		4 (12)	4 (8)
30 - 34		1 (3)	1 (2)
35		1 (3)	1 (2)
Undetermined	1 (7)	3 (9)	4 (8)
Child Score			
Admission			
Child B	9 (60)	4 (12)	13 (26)
Child C	5 (33)	24 (71)	29 (59)
Undetermined	1 (7)	6 (18)	7 (14)
Outcome			
Child B	9 (60)	1 (3)	10 (20)
Child C	5 (33)	27 (79)	32 (65)
Undetermined	1 (7)	6 (18)	7 (14)

(n=49); * Rounded to one digit; **Data are mean (SD).
